# The influence of hay steaming on clinical signs and airway immune response in severe asthmatic horses

**DOI:** 10.1186/s12917-018-1636-4

**Published:** 2018-11-15

**Authors:** Marie Orard, Erika Hue, Anne Couroucé, Céline Bizon-Mercier, Marie-Pierre Toquet, Meriel Moore-Colyer, Laurent Couëtil, Stéphane Pronost, Romain Paillot, Magali Demoor, Eric A. Richard

**Affiliations:** 1 LABÉO Frank Duncombe, Caen, France; 2 Normandie Université, UniCaen, BIOTARGEN, Caen, France; 3 LUNAM Université, Oniris, NP3, Nantes, France; 4 Royal Agricultural University, Cirencester, UK; 5 Purdue University, College of Veterinary Medicine, West Lafayette, IN USA

**Keywords:** Equine asthma, Hay steaming, Inflammation, Cytology, Cytokines

## Abstract

**Background:**

Avoidance of antigenic stimuli was found to significantly reverse airway obstruction of horses with severe equine asthma (sEA). To date, no published study investigated the influence of steaming hay on lower airway condition of sEA-affected horses. The objectives were to determine the clinical, cytological and cytokine respiratory responses of both sEA and control (CTL) horses experimentally exposed to steamed or dry hay.

**Results:**

A cohort of 6 sEA horses and 6 CTL horses was involved in this field study. On day 0, both groups were fed with steamed hay for 5 consecutive days, followed by a wash-out period of 26 days prior to be fed with dry hay for 5 consecutive days. Investigations performed 2 days prior to and 5 days after each challenge included clinical score, tracheal mucus accumulation, and bronchoalveolar lavage fluid (BALF) cytology and cytokine mRNA expression. Feeding steamed hay significantly decreased its mould content (*P* < 0.001). Mucus score significantly increased when feeding dry hay (*P* = 0.01). No significant influence of challenge type was found on clinical score. Percentages of neutrophils (*P* < 0.001) as well as mRNA expression of IL-1β (*P* = 0.024), IL-6R (*P* = 0.021), IL-18 (*P* = 0.009) and IL-23 (*P* = 0.036) in BALF of sEA affected horses were significantly increased after both (steamed and dry hay) challenges. Relative mRNA expression of IL-1β, IL-6R and IL-23 in BALF were also significantly correlated to neutrophil percentages and both clinical and tracheal mucus score.

**Conclusions:**

Steaming significantly decreased mould content but inconsistently influenced the respiratory response of sEA affected horses when fed hay. Based on BALF cytology and cytokine profiles, its relevance might be controversial as a non-medicinal therapy for sEA-affected horses.

**Electronic supplementary material:**

The online version of this article (10.1186/s12917-018-1636-4) contains supplementary material, which is available to authorized users.

## Background

Severe equine asthma (sEA; previously known as recurrent airway obstruction) is a chronic disease of adult horses characterised by frequent coughing and increased respiratory effort at rest, as well as marked lower airway inflammation and reversible airway obstruction [1]. While neutrophils involvement in the pathophysiology of this disease is well defined, controversies still persist in terms of T-helper (Th)-1 and/or Th-2 polarisation, as determined by cytokines mRNA expression in BALF-derived cells [2, 3]. Involvement of Th-17 pathway [4, 5] and regulatory T cells (Treg; [6]) have also been suggested. Exposure to airborne environmental antigens is central in both initiation and maintenance of the disease, especially through stabling and exposure to hay and straw [7]. Feeding mouldy hay indeed represents a long-established model for sEA exacerbation in susceptible horses [2], and also allows differentiation from mild/moderate equine asthma (mEA; previously known as inflammatory airway disease) [3]. Inhalation challenges with aqueous mould extracts were previously found to induce neutrophilic inflammation in sEA affected horses but not in controls [8]. Results from experimental exposure of sEA affected horses to hay dust suspensions highlighted the synergistic effect of endotoxins and other dust components [9].

Environmental change, especially pasture turnout, with or without anti-inflammatory medication was found to significantly reverse airway obstruction of sEA affected horses [10]. On the other hand, corticosteroid therapy while maintaining the horse in a dusty environment leads to improved clinical signs and lung function but persistent airway inflammation [11, 12]. Feeding haylage and bedding wood shavings have been associated with significantly lower levels of both respirable dust (< 5 μm diameter) and endotoxins, within the horse’s breathing zone, when compared to hay/straw environment [13, 14]. Alternatively, soaking and high-temperature steaming of hay for 16 h and 50 min respectively, significantly reduced respirable particles exposure; however endotoxin levels were not investigated [15, 16]. Steaming was also found to be effective at reducing mould content in hay, unlike soaking hay for 9 h [17]. Steaming hay indeed aims to reach and uniformly diffuse a temperature of 100 °C within the hay bale. To date, there is no published study that has investigated the potential influence of steaming hay on lower airway inflammation of sEA-affected horses.

The aim of the study was to determine clinical, cytological and cytokine respiratory responses of both sEA and control (CTL) horses experimentally exposed to steamed or dry hay. Our hypothesis was that feeding sEA affected horses with dry hay will result in higher respirable dust exposure, airway inflammation and clinical signs, as compared to feeding high-temperature steamed hay. Steaming hay indeed significantly decreased mould content but inconsistently influenced the respiratory response of sEA-affected horses. Both cytological and cytokine profiles actually revealed a significant airway inflammation when feeding hay (steamed or not).

## Results

One sEA horse exhibited fever during the wash-out period and was subsequently excluded from the study. In the end, 5 sEA horses and 6 CTL horses completed both challenges (Additional file 1).

### Hay and air sampling

Both bacterial and mould content were significantly lower (*P* < 0.001) in steamed hay (195 280 ± 465 270 and 1 507 ± 1 864 CFU/g, respectively), when compared to dry hay (4 042 000 ± 6 748 619 and 118 150 ± 187 624 CFU/g, respectively; Additional file 2). *Aspergillus* family (principally *A. glaucus* and *A. nidulens*) was identified in 9/10 samples of dry hay and 10/10 samples of steamed hay; *Aspergillus fumigatus* has been identified in one sample of dry hay only. Similarly, *Penicillium* spp. was identified in 3/10 samples of dry hay and 1/10 samples of steamed hay. Concentrations of respirable dust collected within the breathing zones (CTL and sEA horse) were 0.0015 and 0.0034 mg/m^3^, respectively, during challenge #1 (steamed hay) vs. 0.0024 and 0.0057 mg/m^3^ during challenge #2 (dry hay). Similarly, the inhalable fractions collected were 0.0045 and 0.0082 mg/m^3^, respectively, during challenge #1 vs. 0.31 and 0.26 mg/m^3^ during challenge #2. Endotoxin activities from respirable fraction were 0.08 and 0.98 EU/mL, respectively, during challenge #1, vs. 2.19 and 3.88 EU/mL during challenge #2. Levels of β-D-glucan were 340 and 1067 pg/mL, respectively, during challenge #1, vs. 895 and 2367 pg/mL during challenge #2.

### Clinical score and tracheal mucus

**Fig. 1:**
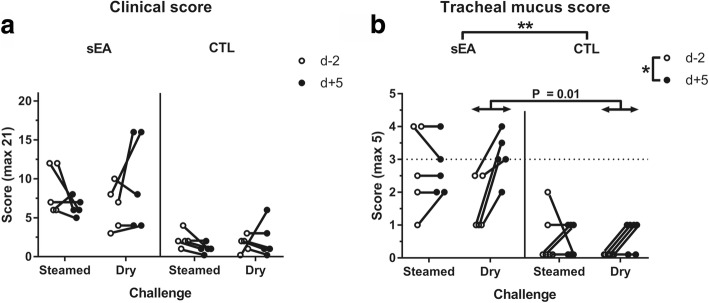
**a** Clinical score and **b** tracheal mucus score, before and after the initiation of each challenge. sEA, severe equine asthma; CTL, control; d-2, 2 days before challenge; d + 5, 5 days after challenge. *, ** significant difference between groups (*P* < 0.05 and < 0.01), based on ANOVA investigation. Significant overall interactions (*P* < 0.05) were also found between ‘challenge’ (steamed, dry) and ‘time’ (d-2, d + 5); the p-value correspond to the subsequent Tukey-Kramer’s post-hoc test

Overall, the mean clinical scores were not significantly different between sEA and CTL horses (‘disease status’; *P* = 0.071) and no significant influence on clinical scores was found for either ‘challenge’ or ‘time’ (Fig. 1), controlling for age. A significant interaction (*P* = 0.049) was found for clinical score between ‘challenge’ (steamed vs. dry hay) and ‘time’ (d + 5 vs. d-2); the post-hoc tests were however not significant for any further comparison. Mean mucus score was significantly higher (*P* = 0.005) for sEA horses when compared to CTL horses and overall significantly higher (*P* = 0.024) at d + 5 when compared to d-2 (Fig. 1). A significant interaction (*P* = 0.022) was also found for tracheal mucus between ‘challenge’ and ‘time’; the score being significantly higher for dry hay at d + 5 when compared to d-2 (*P* = 0.01), but not significantly different between d + 5 and d-2 for steamed hay (*P* = 0.98).

### BALF cytology

**Fig. 2:**
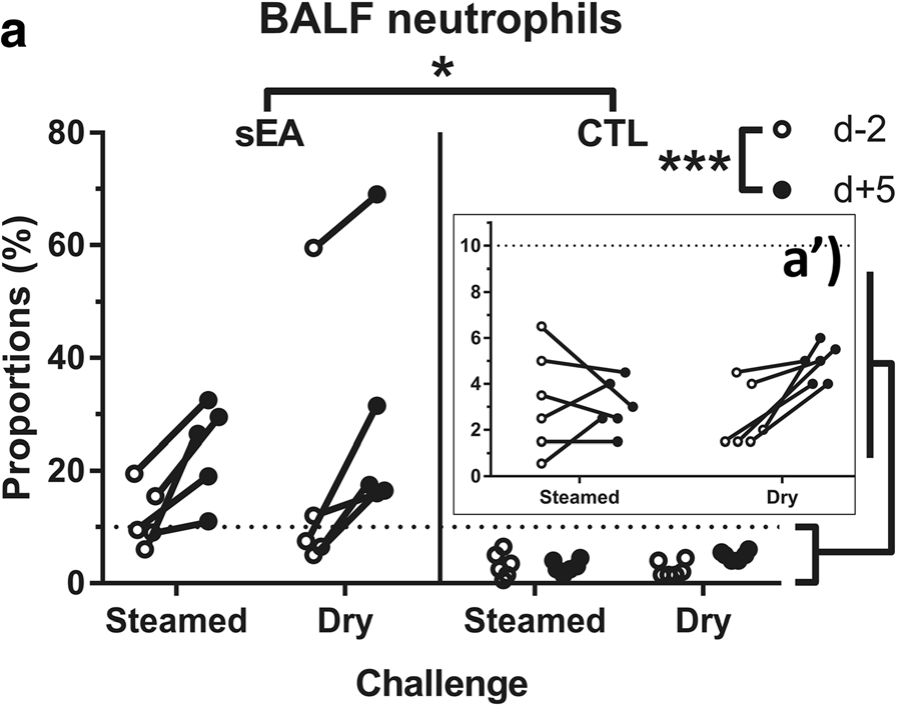
**a**) Mean neutrophil percentages in bronchoalveolar lavage fluid (BALF), before and after the initiation of each challenge; a’) focus (same data) on the control horses. sEA, severe equine asthma; CTL, control; d-2, 2 days before challenge; d + 5, 5 days after challenge. *, *** significant difference between groups or timepoints (*P* < 0.05 and 0.001, respectively), based on ANOVA investigation

After controlling for age, no significant influence on the proportions of BALF volume recovered was found for either ‘challenge’, ‘time’ or ‘disease status’, nor any interaction between categories. Total cell counts were overall significantly higher (*P* = 0.037) for sEA compared to CTL horses (Additional file 3). The mean percentage of BALF neutrophils was significantly higher (*P* = 0.011) for sEA horses when compared to CTL horses and was overall significantly higher (*P* < 0.001) at d + 5 when compared to d-2 (Fig. 2), while no significant interaction was found between ‘challenge’ and ‘time’ (*P* = 0.197). The mean percentage of BALF lymphocytes was significantly lower (*P* = 0.001) at d + 5 when compared to d-2. A significant interaction (*P* = 0.009) was found between ‘disease status’ and ‘time’ for the percentage of BALF macrophages; the post-hoc tests being however not significant. The mean percentage of BALF metachromatic cells were significantly lower (*P* = 0.001) for sEA horses when compared to CTL horses (Additional file 3). No overall influence of the ‘challenge’ type (steamed vs. dry hay), or any interaction between ‘challenge’ and either ‘time’ or ‘disease status’ was found for any of the investigated cytological parameters.

### BALF cytokines

**Fig. 3:**
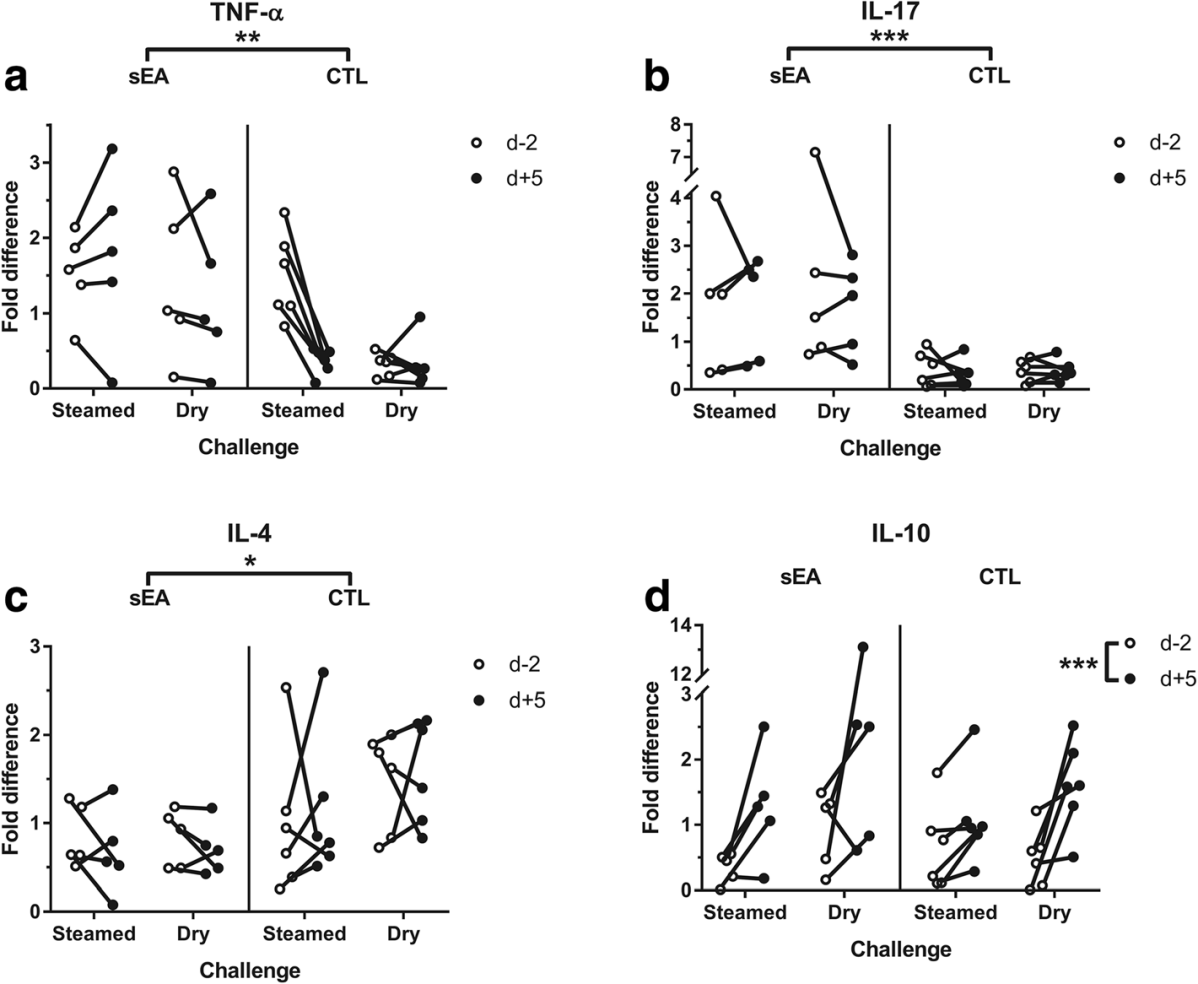
Relative mRNA expression of cytokines in bronchoalveolar lavage fluid (BALF), before and after the initiation of each challenge: **a** tumour necrosis factor (TNF)-α; **b** interleukin (IL)-17; **c** IL-4; **d** IL-10. sEA, severe equine asthma; CTL, control; d-2, 2 days before challenge; d + 5, 5 days after challenge. *, **, *** significant difference between groups or timepoints (*P* < 0.05, 0.01 and 0.001, respectively), based on ANOVA investigation

**Fig. 4:**
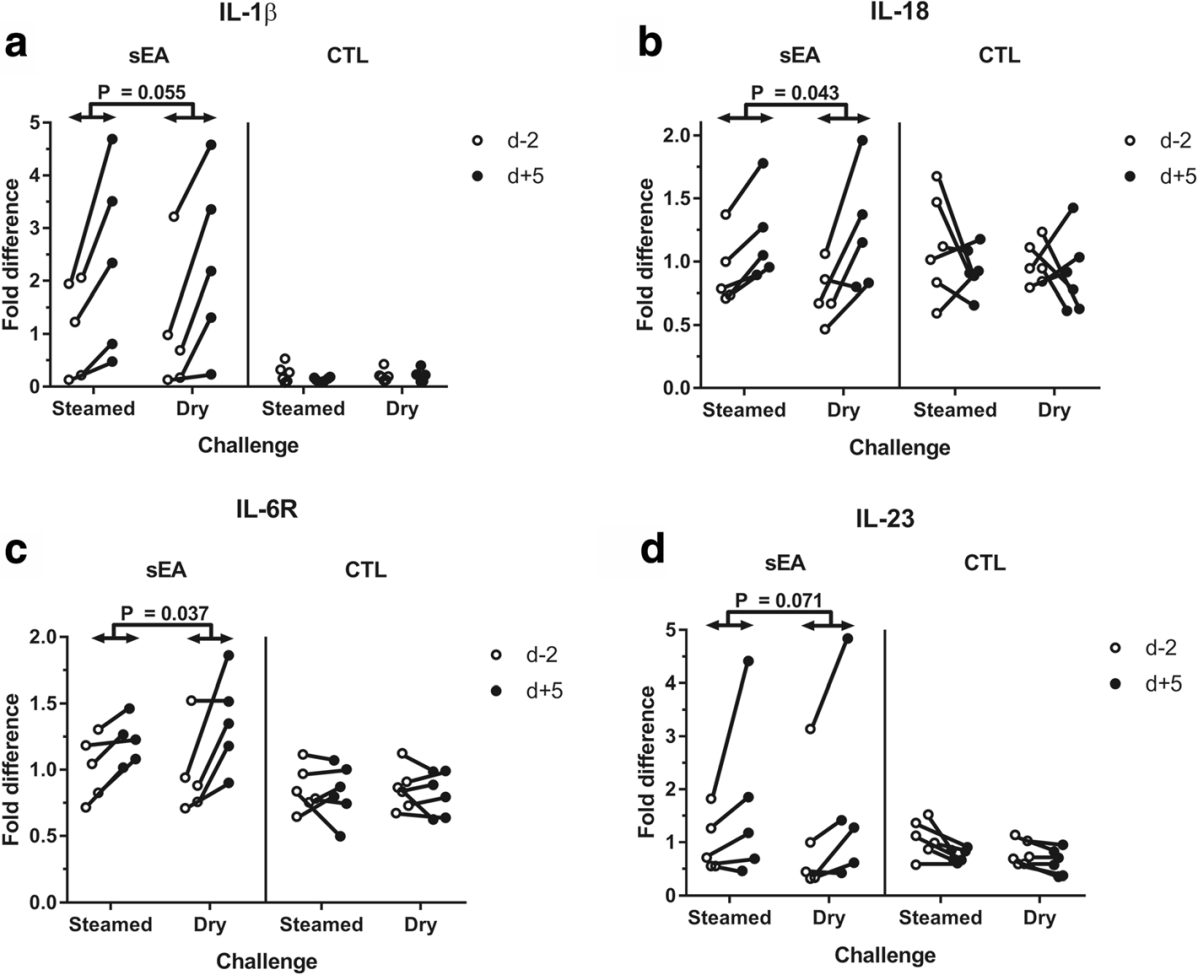
Relative mRNA expression of cytokines in bronchoalveolar lavage fluid (BALF), before and after the initiation of each challenge: **a** interleukin (IL)-1β; **b** IL-18; **c** IL-6R; **d** IL-23. sEA, severe equine asthma; CTL, control; d-2, 2 days before challenge; d + 5, 5 days after challenge. Significant overall interactions (*P* < 0.05) were found between between ‘disease status’ (sEA, CTL) and ‘time’ (d-2, d + 5); the p-values correspond to the subsequent Tukey-Kramer’s post-hoc tests

After controlling for age, mean mRNA expression was significantly lower for IL-4 (*P* = 0.022) and significantly higher for IL-17 (*P* < 0.001) and TNF-α (*P* = 0.006) in BALF of sEA affected horses, when compared to CTL horses (Fig. 3). Overall mRNA expression of IL-10 was significantly higher (*P* < 0.001) at d + 5 when compared to d-2, irrespective of the horse disease status (Fig. 3). Significant interactions were found between ‘disease status’ and ‘time’ (higher at d + 5 than d-2 for sEA horses only) for relative mRNA expression of IL-1β (*P* = 0.024), IL-6R (*P* = 0.021), IL-18 (*P* = 0.009) and IL-23 (*P* = 0.036) in BALF (Fig. 4). No significant variation was found throughout challenges for relative mRNA expression of IL-2, IL-5, IL-6, IL-8, IL-13, IFN-γ and TGF-β (Additional file 4). No overall influence of the ‘challenge’ type (steamed vs. dry hay), or any interaction between ‘challenge’ and either ‘time’ or ‘disease status’ was found for any cytokine relative expression in BALF.

### Correlations

**Fig. 5:**
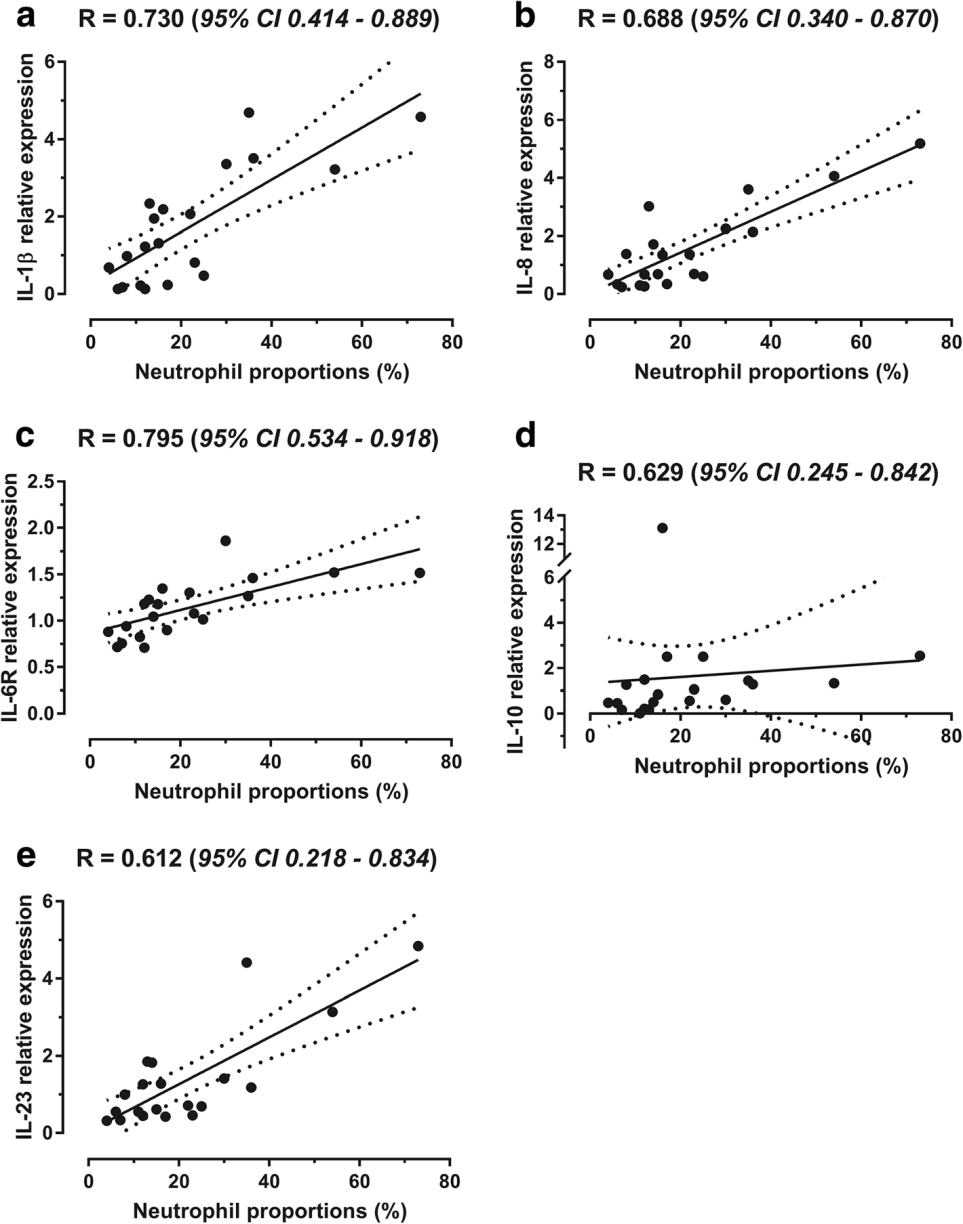
Correlations (95% confidence interval) between relative mRNA expression of cytokines and neutrophil proportions in bronchoalveolar lavage fluid (BALF) of sEA affected horses, before and after the initiation of each challenge: **a** interleukin (IL)-1β; **b** IL-8; **c** IL-6R; **d** IL-10; **e** IL-23. Straight line, linear regression; dotted lines, 95% confidence

Relative mRNA expression of IL-1β, IL-6R, IL-8, IL-10 and IL-23 were significantly associated with BALF neutrophil percentages of sEA affected horses (Fig. 5), and mRNA expression of IL-1β, IL-6R, IL-8, IL-13, IL-23 and TGF-β in BALF were also significantly correlated with tracheal mucus score (Additional file 5). Relative mRNA expression of IL-6R was negatively correlated with the percentage of macrophages in BALF of sEA affected horses, and IL-4 was significantly correlated with metachromatic cell percentages (Additional file 5).

## Discussion

This is, to our knowledge, the first study in which the inflammatory and immune airway responses of both sEA and CTL horses have been investigated through a controlled exposition challenge with steamed and dry hay. Horses with sEA had been on pasture for at least 6 weeks prior to the challenge initiation. Unsurprisingly and due to the persistent nature of this syndrome, some level of clinical and/or cytological abnormalities at the time of enrolment was observed for 2 out of the 5 sEA-affected horses. Interestingly, the initial clinical scores were indeed 12/21 for both horses, and decreased to 6/21 at the end of the first exposure period (to steamed hay). Despite a significant interaction between ‘challenge’ (steamed vs. dry hay) and ‘time’ (d + 5 vs. d-2), the individual variability of clinical scores may have confounded the results. However, 4 out of the 5 sEA horses showed significantly (*P* = 0.01) elevated tracheal mucus score (≥ 3) after being fed dry hay, while the score remained < 3 for a majority of them (3 out of 5) after being fed steamed hay.

Percentages of neutrophils in BALF were overall higher at d + 5 when compared to d-2, irrespective of ‘challenge’ (steamed and dry hay) and ‘disease status’ (sEA and CTL). Induction of a significant neutrophilic airway inflammation has been previously reported in CTL horses through environmental challenges [18, 19]. In the present study, all BALF neutrophil percentages measured from CTL horses systematically remained < 10%, while the same “good quality” (non-mouldy) hay was used in both challenges. Steaming hay significantly decreased mould content by a hundred fold, as previously described [17]. The mould content in hay was investigated in the present study by mycological culture only; the presence of spores, antigenic fragments or mycotoxins has not been evaluated. Targeted and non-targeted methodologies, such as qPCR and Maldi-Tof mass spectrometry, respectively, would considerably increase both the sensitivity and broadness of aeroallergen detection.

Based on the inflammatory responses measured after both challenges, it might be hypothesised that other non-evaluated stimuli might still be present within the hay even after steaming (e.g. mould and bacterial wall fragments). Horses were kept in paddocks during exposure periods, in order to avoid the influence of environmental confinement [20] and associated management of stables ventilation. Alternatively, steaming hay may not have been sufficient to prevent the synergistic inflammatory activity of various hay dust components, especially including endotoxins, which has previously been demonstrated experimentally [9]. When considering air sampling within the breathing zone during the present study, the inhalable fraction was 100 times lower when feeding steamed hay compared to dry hay. The influence of steaming hay on the respirable particles was however more limited in the current study, while this fraction was found to be significantly associated with lower airway inflammation of horses [21]. Respirable dust, endotoxin and β-D-glucan levels may also have been affected by individual horse’s behaviour when eating hay, as previously demonstrated for particulate concentrations [22]. The mean values measured for endotoxin activities within the breathing zones were 5.7 times lower with steamed hay, when compared to dry hay, while a large variability was observed among horses. Interestingly, values for endotoxin activities within the breathing zone when feeding steamed hay were also comparable with previously published data for haylage/wood shavings combination [14].

No significant influence of the ‘challenge’ type (steamed vs. dry hay) was found for any cytokine mRNA expression in BALF. Significant interactions were found between ‘disease status’ (sEA vs. CTL) and ‘time’ (d + 5 vs. d-2) for mRNA expression of IL-1β and IL-18; relative mRNA expression of TNF-α was also significantly higher in BALF from sEA affected horses, when compared with CTL horses. Such observations are coherent with the measurable inflammatory response of sEA affected horses to both (steamed and dry) hay challenges. Interestingly, mRNA expression of IL-6R in BALF was also increased post-challenges for sEA affected horses only. To date, the relative expression of this receptor has not been investigated in BALF of horses. Interestingly, a genome-wide association study identified a significant association between a single nucleotide polymorphism in the IL-6R gene and the risk of asthma in humans [23]. Further investigations are warranted to characterize the IL- 6 signaling pathway involvement in equine asthma, especially in terms of subsequent T-helper (Th) polarisation as recently demonstrated in humans [24].

The previously suggested involvement of Th17 pathway in the airways of horses with severe asthma [4, 5], is supported by the present study. Overall IL-17 mRNA expression was indeed significantly higher in BALF of sEA affected when compared with CTL horses. The IL-23 mRNA expression in BALF was also increased post-challenges for sEA affected horses only, and was furthermore significantly correlated with both tracheal mucus score and BALF neutrophil percentages. Surprisingly, IL-8 mRNA expression was neither significantly different among groups (*P* = 0.11; controlling for age of the horses) nor significantly influenced by the challenge types. However, a significant correlation (*R* = 0.688) was found with neutrophil percentages in BALF. While overexpression has been associated with sEA chronicity [4, 5], it is likely that IL-8 contributes to neutrophil chemotaxis in the earlier stages of equine asthma [2]. The short duration of each challenge period in the current study might partly explain the limited amplitude of IL-8 mRNA expression in BALF.

The lower mRNA expression of IL-4 in BALF of sEA versus CTL horses was unexpected, mainly based on the previously demonstrated Th2 polarisation for this syndrome [25]. Interestingly, mRNA expression of IL-4 in BALF of both sEA and CTL horses was also not significantly influenced by either challenge. The sEA group exhibited significantly lower percentages of metachromatic cells in BALF, when compared to CTL horses; and IL-4 mRNA expression was significantly correlated with both eosinophil and metachromatic cell percentages. When considering a possible Treg involvement in sEA, mRNA expression of TGF-β was interestingly not significantly modified throughout the study. On the other hand, IL-10 was the only cytokine which mRNA expression in BALF was significantly higher post-challenges and correlated to BALF neutrophil proportions, with however no significant difference between sEA and CTL horses. Surprisingly, mRNA expression of IL-10 in BALF from sEA affected horses was previously found not to be significantly influenced by various environmental challenges [26]. Alternatively, percentages of different Treg subpopulations, as characterised by flow cytometry, were found to be significantly higher in BALF of sEA horses during crisis exacerbation when compared to remission [6].

### Study limitations

All 12 horses involved in this field trial were client-owned, with differences in terms of geographic origins prior to enrolment in the study. The limited number of horses enrolled in this study was consistent with similar works previously published [4, 8]. Based on the current results, the retrospective statistical power analyses highlights the variability of sample size required in relation to the multiple outcomes investigated. Values of power indeed ranged from 30% for clinical score to 82 and 98% for respectively mucus score and BALF neutrophil percentages. Unlike mucus and cytology (for which estimated sample size = 4–6), a minimum of 20 horses would have been required for clinical signs to reach a 80% power with a α-level of 0.05. The average age was significantly different between groups; the CTL horses being significantly younger than the sEA affected horses. This confounding factor was then taken into account in all further statistical analyses. In terms of design, both challenges (steamed and dry hay) were not performed as a cross-over study, since horses were initially fed with steamed hay (challenge #1) and subsequently fed dry hay (challenge #2). For ethical reasons, the design (i.e. steamed then dry hay) aimed at limiting the risk of horse exclusion (due to crisis exacerbation and subsequent treatment) at the end of the first exposure period, which would have precluded the horse to be involved in the second period. No significant difference was found for each group at d-2 (i.e. initiation of each exposure period) for all investigated parameters; suggesting that horses returned to “baseline level” prior to challenge #2. Also, clinical and tracheal mucus scoring were performed by clinicians not blinded to either challenge type (steamed vs. dry) or horse disease status (sEA vs. CTL), unlike all subsequent laboratory analyses, and lung function tests were not available for this study. Finally, the immune responses in BALF were investigated for the various cytokines through relative mRNA expressions only. Measuring BALF cytokine concentrations using commercially available ELISA kits however lacks sensitivity, since many BALF samples previously showed undetectable cytokine concentrations [27, 28].

## Conclusions

Steaming hay significantly decreased mould content and induced less tracheal mucus in horses. However, both types of hay (steamed or not) induced BALF neutrophilia, and none induced respiratory clinical signs. The relevance of steaming hay however warrants further investigations both in the context of prevention/therapy for mEA horses at training and for long-term preclusion of lately developing sEA.

## Methods

### Horses

A cohort of 12 privately owned leisure horses were used in the study, and was subdivided into 2 separate groups. The control (CTL) group included 6 young horses (4 mares and 2 geldings, aged 2–5 years). These horses had no history of coughing or nasal discharge, exhibited no tracheal mucus upon endoscopy, and had less than 10% neutrophils in bronchoalveolar lavage fluid (BALF) from each lung when investigated 10 days prior to the trial. The other group included 6 adult horses (5 mares and 1 gelding, aged 9–17 years) that had all previously been diagnosed by the same clinician as suffering from sEA, based on clinical investigations, BALF cytology and reversible airway obstruction after medical/environmental change. Only horses kept in pasture and without any medical treatment or access to hay for at least 1 month prior to the trial were enrolled in the study. Recruitment for the study was made on a volunteer basis from private training centres (CTL horses) and from the caseload previously referred to the hospital (sEA horses; Oniris, Nantes). All owners signed an informed consent, and all procedures were performed by veterinarians and complied with relevant guidelines (Directive 2010/63/EU). The study was also approved by the Institutional Ethic Committee for Clinical Research (CERVO-2017-8-V).

### Study design

**Fig. 6:**
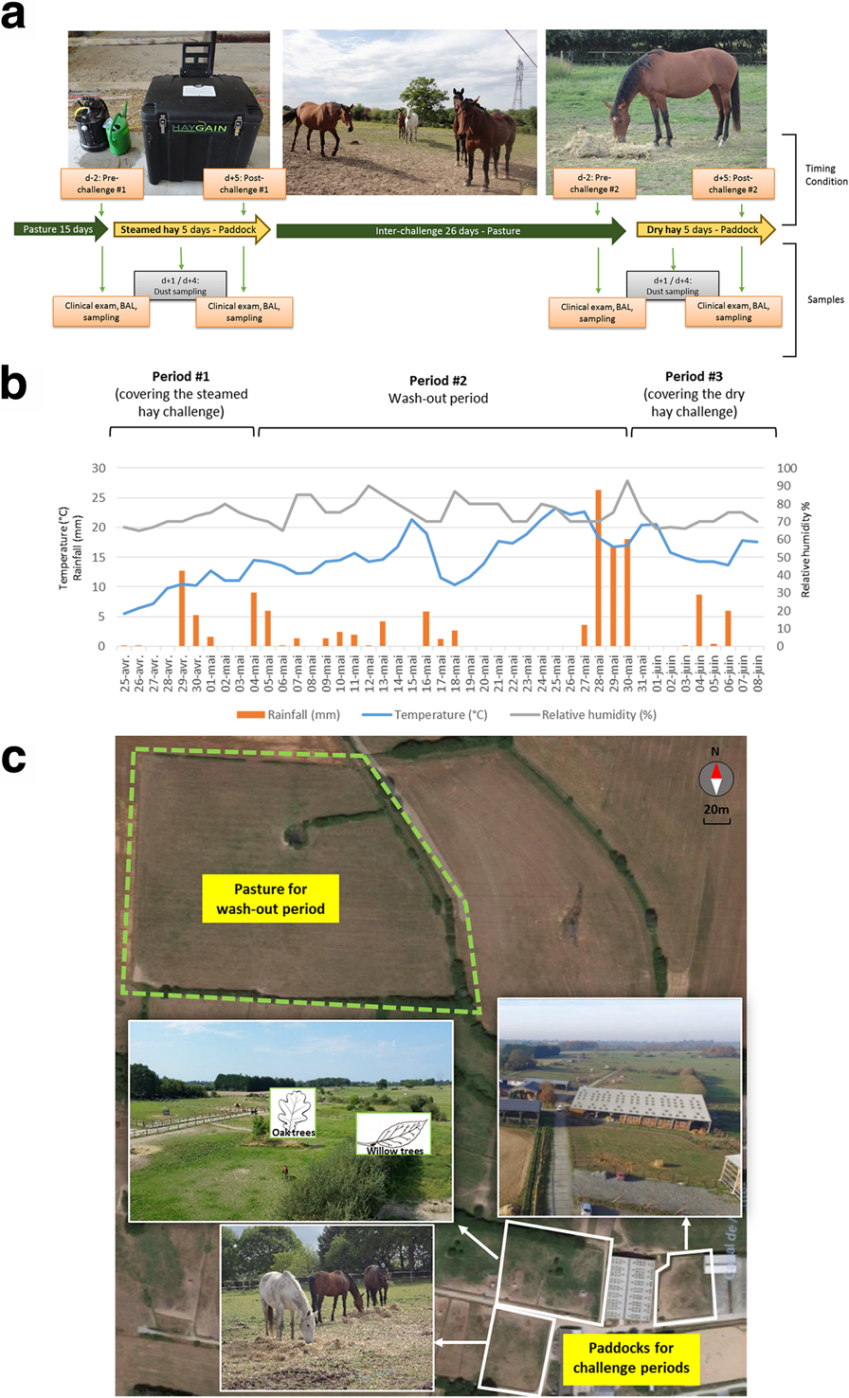
Overview of the study: **a** experimental design, **b** climatic conditions, and **c** geographical repartition

This study was conducted during spring 2017. At the initiation of the trial, all horses were gathered and kept in a 4 ha pasture without access to hay for a duration of 2 weeks (Fig. 6). Since stabling was previously found to be associated with increased airway inflammation [29], horses were kept in 1 000 m^2^ paddocks by subgroups of three individuals during both exposure periods. On day 0 of the trial, both groups (sEA and CTL) were fed on the ground with about 5 kg per horse of steamed hay (challenge #1), twice daily for 5 consecutive days (i.e. exposure period #1); followed by a wash-out period of 26 days in the 4 ha pasture without access to hay, prior to a second challenge with similar distribution of dry hay (challenge #2) for 5 consecutive days, (exposure period #2). Horses had access to water ad libitum and were also fed flaked cereals and dry pellets (Twenty Horse^1^) twice daily. Good quality (non-mouldy) grass hay was harvested as square bales (12–15 kg). For the challenge #1, the hay was steamed using a commercial device (HG-600^2^), according to manufacturer’s recommendations, and immediately distributed to horses. Briefly, the hay bale was placed onto the steamer and pushed down firmly until the manifold spikes pierce the hay to their full length; the steam generator (containing water) was then activated and steam diffused in hay through the spikes. At the end of exposure period #2, asthmatic horses received dexamethasone (0.1 mg/kg IV for 2 days, then progressively decreasing doses over 1 week) and all horses (sEA and CTL) were returned to their owners. Two days before (d-2) and 5 days after (d + 5) the initiation of each challenge, all horses underwent a full clinical evaluation, as well as a tracheal endoscopy and BALF sampling. The 2010 CONSORT guidelines for reporting clinical trial were used for this report (Additional files 1 and 6) [30, 31].

### Hay characterisation

Hay samples (random sampling by hand for a total of 1 kg) were collected from 10 different square bales before and after the steaming procedure, and stored in dedicated bags^1^. All square bales originated from the same batch of hay (one single pasture), and all analyses were performed immediately after sampling. For bacteriology, 20 g of crushed hay were mixed with 180 ml of 0.1% peptone water and diluted 1/10 up to 10^− 6^. One ml of each dilution was plated onto a Plate Count Agar (PCA) and incubated at 37 °C. One ml of each dilution was also cultured in oxytetracycline-glucose-yeast Extract Agar (OGA) and incubated at 25 °C for fungal growth. Bacterial and fungal growths were expressed as colony forming units (CFUs) after 5 days of incubation. The fungal colonies were then isolated on Sabouraud agar in order to be identified.

### Air quality

Air sampling within the breathing zone was performed continuously for 2-3 h during hay feeding, on d + 1 (one CTL horse) and d + 4 (one sEA horse) of each exposure period. Respirable and inhalable particulates were collected according to previously published procedures [21].

### Particulates

Horses were equipped with two samplers. The respirable fraction was collected with a flow rate of 2.5 L/min (AirCheck XR5000^3^) onto 37 mm type AE glass fibre filters using aluminium cyclone. The inhalable fraction was collected with a flow rate of 2.0 L/min (AirCheck 3000^3^) onto 25 mm PVC filters using Institute of Occupational Medicine (IOM) personal sampler. Sampling pumps were calibrated (Defender Bios calibrator^3^) before and after sampling. Measurements of respirable and inhalable dust were assessed by gravimetric method, in triplicate measurements. The weight of particulates was divided by volume of air sampled to obtain airborne concentrations. Filters were placed in a desiccator for 24 h prior to any weight measurement. Negative controls were similarly prepared and only transported to and from the site of trial.

### Endotoxins

Endotoxins were measured from the 37 mm type AE glass fibre filters, which were kept frozen (− 20 °C) after particulates measurements. Filters were eluted in 10 mL of 0.05% Tween 20^4^ within pyrogen-free water. Samples were then shaken during 1 h (MS-NRK-30^5^). After elution, the suspension was centrifuged (1000 *g*; 15 min). The supernatant was then kept frozen (− 20 °C) until being analysed. The eluates provided from sample filters were diluted at 1/10 in pyrogen-free water. Endotoxin activities were assayed using Endosafe – PTS2005F,^6^ according to manufacturer’s recommendations.

### β-D-glucan

Following endotoxin elution, heat extraction was performed in order to measure (1–3)-β-D-glucan content. Samples were then respectively diluted 10-fold for blank filters and 100-fold for sample filters in pyrogen-free water. Measures were carried out in duplicates, using a Glucatell test,^7^ according to manufacturer’s recommendations. The range of quantification was 5–40 pg/mL.

### Clinical score, tracheal mucus and bronchoalveolar lavage fluid cytology

On d-2 and d + 5 for each exposure period, a clinical score, based on a 0–21 scale (Additional file 7) [32], was assigned to each horse. Detomidine hydrochloride (0.01 mg/kg IV) and butorphanol tartrate (0.01 mg/kg IV) was given prior to endoscopy. Airway endoscopy and BAL collection were performed using a flexible 3.2 m long, 12.8 mm tip diameter videoendoscope.^8^ The amount of tracheal mucus was scored (grade 0–5) according to the previously published scale [33]. Left and right lungs were then sampled according to previously published procedures [34], with minor modifications. Briefly, a total of 500 mL of warmed isotonic saline solution (2 boluses of 250 mL each) was instilled and aspirated for each lung. Samples were conditioned (+ 4 °C in EDTA tubes) and processed (cytocentrifugation, May-Grünwald-Giemsa staining) as described previously [35]. Differential cell counts were performed on 300 leukocytes. Unlike clinical and mucus scores, cytological investigations of BALF from both lungs were conducted by investigators blinded to challenge type and horse status.

### RNA isolation and reverse transcription

Each BALF sample obtained from the right lung was conditioned in order to determine cytokine relative expressions. Expression of 15 cytokines (representative of the different pathways previously associated with sEA) and one receptor (described in human allergic asthma) have been targeted in this study: interferon (IFN)-γ, interleukin (IL)-1β, IL-2, IL-4, IL-5, IL-6, IL-6R, IL-8, IL-10, IL-12, IL-13, IL-17, IL-18, IL-23, transforming growth factor (TGF)-β and tumour necrosis factor (TNF)-α.

Twenty milliliters of BALF was centrifuged and the cell pellets resuspended in RNA protect Cell Reagent.^9^ Total RNA was extracted from cell pellets using the RNeasy Plus Mini Kit^9^. Concentration and purity of total extracted RNA was assessed using a NanoDrop 2000c Spectrophotometer^10^; and RNA integrity was evaluated using a 2100 Bioanalyzer^11^ and the RNA 6000 Nano kit^11^. Mean value (± standard deviation) of RNA integrity number (RIN) for all samples was 9.7 ± 0.2. After extraction, 200 ng was retrotranscribed and cDNA generated using a Superscript Vilo cDNA synthesis kit,^12^ according to the manufacturer’s recommendations. All cDNA samples were stored at − 20 °C until further use. Apart from the cytokines of interest, three reference genes, namely β-actin (ACTB), glyceraldehyde-3-phosphate dehydrogenase (GAPDH) and β-glucuronidase (β-GUS), were also assessed. For each reaction, cDNA was amplified in a 25 μl standard reaction (Taqman Universal PCR Master Mix^10^). Thermal cycling profile used was: 10 min at 95 °C, followed by 50 cycles of 15 s at 95 °C and 1 min at 60 °C. The relative amount of mRNA was calculated using the relative standard curve method (Genex 6.1^13^), and results were expressed as mean fold difference (target / reference genes). Using Normfinder, GAPDH and β-GUS were both selected for normalization (Additional file 8). Molecular investigations were also conducted blinded, in terms of horse ‘disease status’ and ‘challenge’ type.

### Statistical analyses

Continuous data distributions which were not normally distributed, as assessed by Shapiro-Wilk W test, were log-10 transformed. For hay-related parameters, paired *t* tests (steamed vs. dry) were performed for the bacterial/mould content. For all horse-related parameters, effects of challenge (steamed vs. dry), time (d + 5 vs. d-2) and disease status (sEA vs. CTL) were investigated by 3-way analysis of variance (ANOVA, General Linear Model) and Tukey-Kramer’s post-hoc tests, with age as covariate. Correlations among sEA affected horses were determined by Spearman’s correlation coefficient. Absolute values of *R* < 0.5 were arbitrarily not reported. The different analyses were conducted using Prism 7^14^ and NCSS12,^15^ and values of *P* < 0.05 were considered significant.

## Additional files

Additional file 1: CONSORT flow diagram for the clinical trial involving 6 control (CTL) horses and 6 horses with severe equine asthma (sEA). (PDF 46 kb)

Additional file 2: Microbiological content in hay (*n* = 10), before and after steaming: a) bacterial content; b) mould content. *** significantly different *(P < 0.001),* based on paired t test. (TIF 917 kb)

Additional file 3: Cytology of bronchoalveolar lavage fluid (BALF), before and after the initiation of each challenge. sEA, severe equine asthma; CTL control; d-2, 2 days before challenge; d + 5, 5 days after challenge. * overall significant difference between groups (sEA vs. CTL horses); # overall significant difference between time points (d + 5 vs. d-2), based on ANOVA investigations. (DOCX 20 kb)

Additional file 4: Relative mRNA expression of cytokines in bronchoalveolar lavage fluid (BALF), before and after the initiation of each challenge: a) interleukin (IL)-2; b) IL-5; c) IL-13; d) TGF-β; e) IL-6; f) IL-8; g) Interferon (IFN)-γ. sEA, severe equine asthma; CTL, control; d-2, 2 days before challenge; d + 5, 5 days after challenge. (TIF 2119 kb)

Additional file 5: Correlations (95% confidence interval) between relative mRNA expression of cytokines and clinical/cytological parameters, before and after the initiation of each challenge. IL, interleukin; (95% CI); ns, non-significant and/or absolute value of R < 0.5. (DOCX 21 kb)

Additional file 6: CONSORT check-list for the clinical trial involving 6 control (CTL) horses and 6 horses with severe equine asthma (sEA). (PDF 134 kb)

Additional file 7: Clinical scoring system for respiratory conditions, adapted from Tesarowski et al. [32]. (DOCX 18 kb)

Additional file 8: Nucleotide sequences of equine-specific primers used in real-time PCR assays. (DOCX 22 kb)

## Abbreviations

ACTB: Actin
BALF: Bronchoalveolar lavage fluid
cDNA: Complementary deoxyribonucleic acid
CFUs: Colony forming units
CTL: Control
GAPDH: Glycéraldéhyde-3-phosphate dehydrogenase
IFN: Interferon
IL: Interleukin
mEA: Mild equine asthma
mRNA: Ribonucleic acid messenger
OGA: Oxytetracycline-glucose-yeast extract agar
PCA: Plate count agar
qPCR: Quantitative polymerase chain reaction
RIN: RNA Integrity number
sEA: Severe equine asthma
TGF: Transforming growth factor
Th: T-helper
TNF: Tumor necrosis factor
β-Gus: β-glucuronidase

## Footnotes

1 Tromelin & Cie, Ploeren, France

2 Haygain, Lambourn, Berkshire, UK

3 SCK Inc., Eighty Four, PA, USA

4 Merck-Millipore, Molsheim, France

5 Major Science, Saratoga, CA, USA

6 Charles River Laboratories, Saint-Germain-Nuelles, France

7 Associates of Cape Cod Inc., East Falmouth, MA, USA

8 Optomed, Les Ulis, France

9 Quiagen, Courtabœuf, France

10 Thermoscientific, Villebon-sur-Yvette, France

11 Agilent Technologies, Les Ulis, France

12 Life Technologies, Saint-Aubin, France

13 bioMMC, Freising, Germany

14 GraphPad, La Jolla, CA, USA

15 NCSS – LLC, Kaysville, UT, USA
