# Supplementary material for: The influence of hay steaming on clinical signs and airway immune response in severe asthmatic horses
Source: BMC Vet Res. 2018 Nov 15;14:345. doi: 10.1186/s12917-018-1636-4 (PMC6236910; doi:10.1186/s12917-018-1636-4)

CONSORT

TRANSPARENT REPORTING of TRIALS

CONSORT 2010 Flow Diagram: The influence of hay steaming on clinical signs and airway inflammation in severe asthmatic horses.

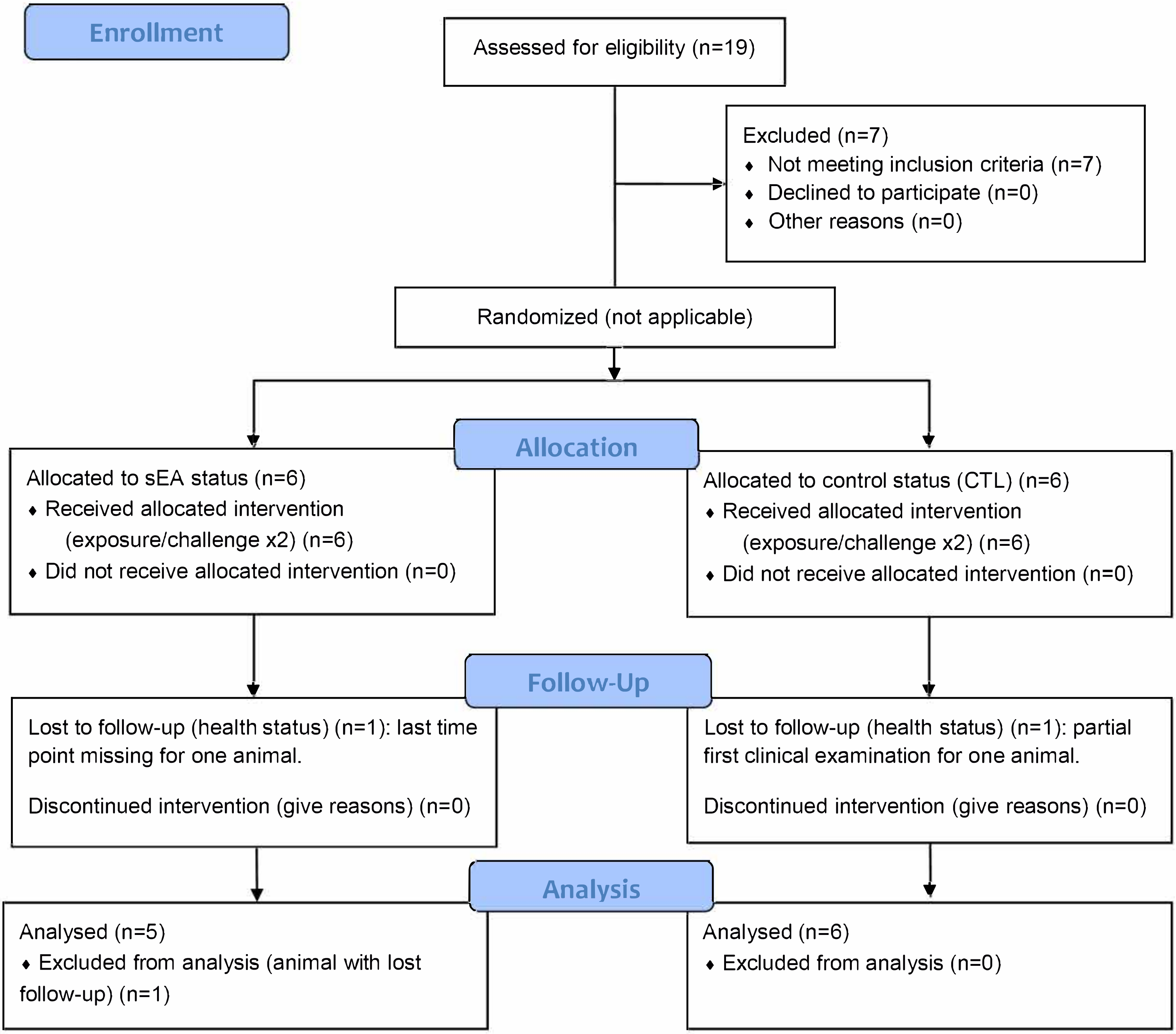

Supplement: Supplementary file 1 — CONSORT flow diagram for the clinical trial involving 6 control (CTL) horses and 6 horses with severe equine asthma (sEA). (PDF 46 kb) [file 12917_2018_1636_MOESM1_ESM.pdf]
