# Supplementary figures and images for: The influence of hay steaming on clinical signs and airway immune response in severe asthmatic horses
Source: BMC Vet Res. 2018 Nov 15;14:345. doi: 10.1186/s12917-018-1636-4 (PMC6236910; doi:10.1186/s12917-018-1636-4)

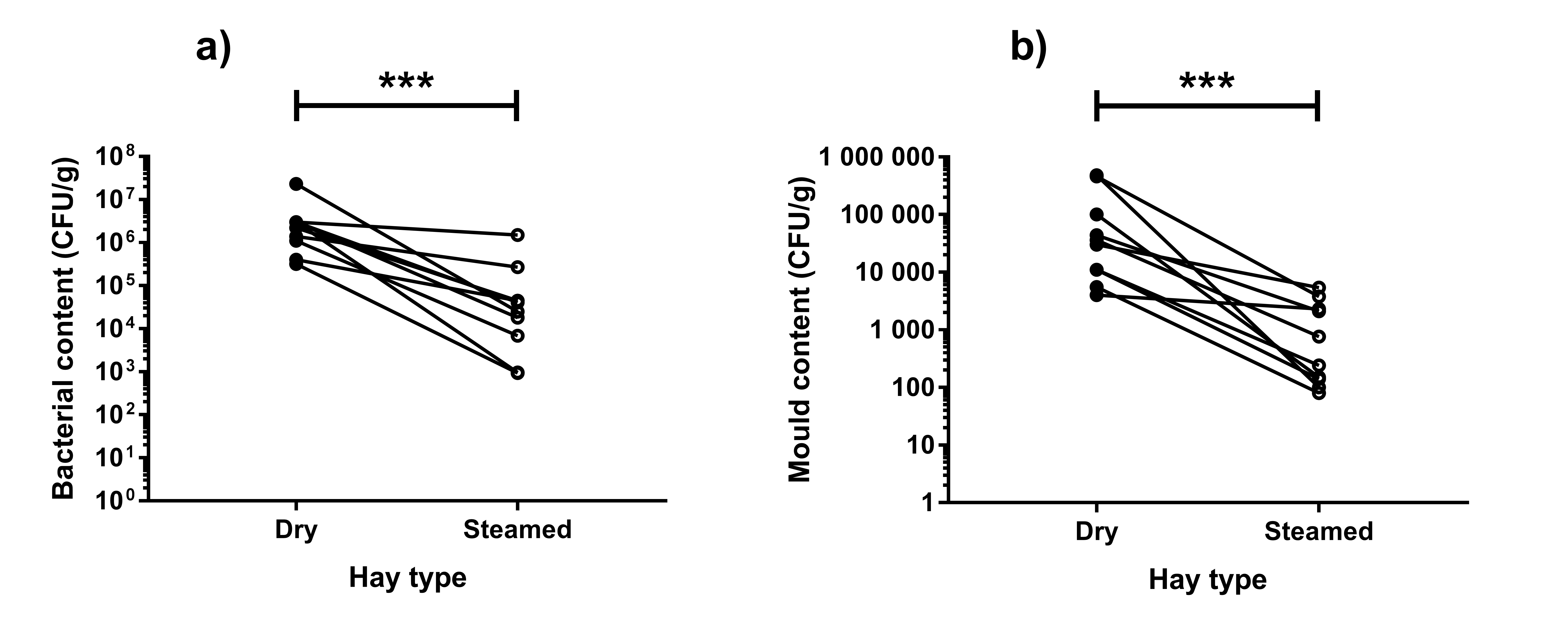

Supplement: Supplementary file 2 — Microbiological content in hay (n = 10), before and after steaming: a) bacterial content; b) mould content. *** significantly different (P < 0.001), based on paired t test. (TIF 917 kb) [file 12917_2018_1636_MOESM2_ESM.tif]

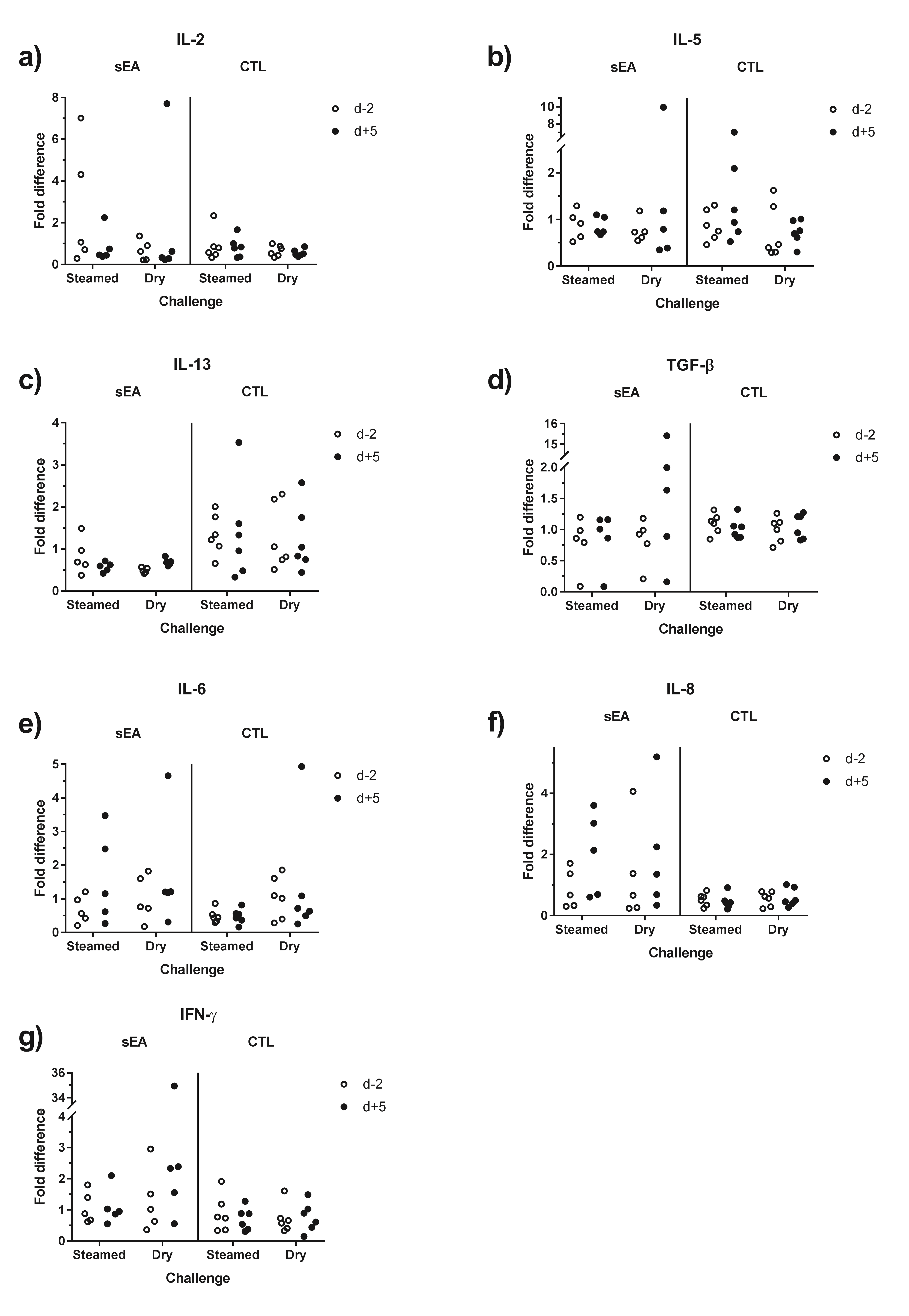

Supplement: Supplementary file 4 — Relative mRNA expression of cytokines in bronchoalveolar lavage fluid (BALF), before and after the initiation of each challenge: a) interleukin (IL)-2; b) IL-5; c) IL-13; d) TGF-β; e) IL-6; f) IL-8; g) Interferon (IFN)-γ. sEA, severe equine asthma; CTL, control; d-2, 2 days before challenge; d + 5, 5 days after challenge. (TIF 2119 kb) [file 12917_2018_1636_MOESM4_ESM.tif]
