# Supplementary material for: The influence of hay steaming on clinical signs and airway immune response in severe asthmatic horses
Source: BMC Vet Res. 2018 Nov 15;14:345. doi: 10.1186/s12917-018-1636-4 (PMC6236910; doi:10.1186/s12917-018-1636-4)
Supplement: Supplementary file 3 — Cytology of bronchoalveolar lavage fluid (BALF), before and after the initiation of each challenge. sEA, severe equine asthma; CTL control; d-2, 2 days before challenge; d + 5, 5 days after challenge. * overall significant difference between groups (sEA vs. CTL horses); # overall significant difference between time points (d + 5 vs. d-2), based on ANOVA investigations. (DOCX 20 kb) [file 12917_2018_1636_MOESM3_ESM.docx]

**Additional file 3: Cytology of bronchoalveolar lavage fluid (BALF), before and after the initiation of each challenge.**

|  | sEA horses | | | | CTL horses | | | |
| --- | --- | --- | --- | --- | --- | --- | --- | --- |
|  | Steamed hay | | Dry hay | | Steamed hay | | Dry hay | |
|  | d-2 | d+5 | d-2 | d+5 | d-2 | d+5 | d-2 | d+5 |
| Recovery  (%) | 42  *(29 – 44)* | 44  *(37 – 48)* | 42  *(33 – 50)* | 38  *(29 – 44)* | 58  *(47 – 65)* | 59  *(51 – 70)* | 58  *(43 – 68)* | 595  *(46 – 64)* |
| Total cell  count * (/mm^3^) | 415  *(285 – 655)* | 250  *(220 – 565)* | 400  *(370 – 460)* | 310  *(230 – 690)* | 320  *(213 – 475)* | 330  *(263 – 428)* | 320  *(210 – 488)* | 450  *(373 – 515)* |
| Metachromatic  cells * (%) | 2.0  *(1.8 – 3.0)* | 1.0  *(0.8 – 1.3)* | 1.0  *(1.0 – 2.0)* | 1.0  *(1.0 – 2.0)* | 2.0  *(1.0 – 3.8)* | 2,5  *(1.3 – 3.8)* | 3.0  *(2.0 – 3.8)* | 2.0  *(2.0 – 2.8)* |
| Eosinophils  (%) | 0.0  *(0.0 – 0.0)* | 0.0  *(0.0 – 0.0)* | 0.0  *(0.0 – 0.0)* | 0.0  *(0.0 – 0.0)* | 0.0  *(0.0 – 0.0)* | 0.0  *(0.0 – 4.3)* | 1.0  *(0.0 – 3.8)* | 2.5  *(1.0 – 6.3)* |
| Macrophages  (%) | 51  *(50 – 54)* | 40  *(34 – 45)* | 40  *(35 – 50)* | 45  *(32 – 48)* | 55  *(51 – 64)* | 56  *(51 – 68)* | 49  *(41 – 64)* | 56  *(51 – 60)* |
| Lymphocytes ^#^  (%) | 36  *(29 – 38)* | 32  *(56 – 45)* | 46  *(29 – 49)* | 32  *(25 – 34)* | 39  *(33 – 43)* | 30  *(26 – 39)* | 41  *(29 – 48)* | 34  *(27 – 36)* |

*sEA, severe equine asthma; CTL control; d-2, two days before challenge; d+5, five days after challenge.* overall significant difference between groups (sEA vs. CTL horses); ^#^ overall significant difference between time points (d+5 vs. d-2), based on ANOVA investigations*
