# Supplementary material for: The influence of hay steaming on clinical signs and airway immune response in severe asthmatic horses
Source: BMC Vet Res. 2018 Nov 15;14:345. doi: 10.1186/s12917-018-1636-4 (PMC6236910; doi:10.1186/s12917-018-1636-4)
Supplement: Supplementary file 5 — Correlations (95% confidence interval) between relative mRNA expression of cytokines and clinical/cytological parameters, before and after the initiation of each challenge. IL, interleukin; (95% CI); ns, non-significant and/or absolute value of R < 0.5. (DOCX 21 kb) [file 12917_2018_1636_MOESM5_ESM.docx]

**Additional file 5: Correlations (*95% confidence interval*) between relative mRNA expression of cytokines and clinical/cytological parameters of sEA affected horses, before and after the initiation of each challenge.**

| Cytokines | Mucus score | Macrophages | Metachromatic cells |
| --- | --- | --- | --- |
| IL1-β | 0.671  *(0.313 – 0.862)* | ns | ns |
| IL-4 | ns | ns | 0.503  *(0.063 – 0.779)* |
| IL-6R | 0.645  *(0.270 – 0.850)* | -0.535  *(-0.795 to -0.107)* | ns |
| IL-8 | 0.607  *(0.212 – 0.850)* | ns | ns |
| IL-13 | 0.537  *(0.110 – 0.797)* | ns | ns |
| IL-23 | 0.756  *(0.459 – 0.901)* | ns | ns |
| TGF-β | 0.675  *(0.319 – 0.864)* | ns | ns |

*IL, interleukin; (95% CI); ns, non-significant and/or absolute value of R < 0.5*
