# Supplementary material for: The influence of hay steaming on clinical signs and airway immune response in severe asthmatic horses
Source: BMC Vet Res. 2018 Nov 15;14:345. doi: 10.1186/s12917-018-1636-4 (PMC6236910; doi:10.1186/s12917-018-1636-4)
Supplement: Supplementary file 7 — Clinical scoring system for respiratory conditions, adapted from Tesarowski et al. [32]. (DOCX 18 kb) [file 12917_2018_1636_MOESM7_ESM.docx]

**Additional file 7: Clinical scoring system for respiratory conditions, adapted from Tesarowski *et al.* (1996) [1]**

| Variable | Descriptor | Score |
| --- | --- | --- |
| Respiratory rate  (breaths / min) | < 15  16 – 20  21 – 25  26 – 29  > 30 | 0  1  2  3  4 |
| Nasal discharge | None  Serous  Mucopurulent | 0  1  3 |
| Abdominal lift | None  Mild (perceptible heave line)  Pronounced (abdomen, thorax  and anal movement) | 0  1  3 |
| Nasal flaring | None  Present | 0  1 |
| Tracheal sounds | Normal (tubular sound)  Increase in intensity  Mucus movement | 0  1  3 |
| Crackles | None  Present | 0  2 |
| Wheezes | None  Present | 0  2 |
| Cough | None  Intermittent  Paroxysmal | 0  1  3 |
| TOTAL |  | / 21 |

1. Tesarowski DB, Viel L, McDonell WN Pulmonary function measurements during repeated environmental challenge of horses with recurrent airway obstruction (heaves). Am J Vet Res. 1996 ;57:1214–1219
