# Supplementary material for: The influence of hay steaming on clinical signs and airway immune response in severe asthmatic horses
Source: BMC Vet Res. 2018 Nov 15;14:345. doi: 10.1186/s12917-018-1636-4 (PMC6236910; doi:10.1186/s12917-018-1636-4)
Supplement: Supplementary file 8 — Nucleotide sequences of equine-specific primers used in real-time PCR assays. (DOCX 22 kb) [file 12917_2018_1636_MOESM8_ESM.docx]

**Additional file 8: Nucleotide sequences of equine-specific primers used in real-time PCR assays**

| **Target** | **Primers and Probe (5'-3')** | **Product**  **size (bp)** | **GenBank**  **Accession** | **References** |
| --- | --- | --- | --- | --- |
| **ACTB** | Forward: AGCGAAATCGTGCGTGACA  Reverse: GCCATCTCCTGCTCGAAGT  Probe: CAAGGAGAAGCTCTGCTATGTCGCCCT | 70 | NM_001081838 | [1] |
| **β-GUS** | Forward: GAAAGGTCTGCTGGAACAGTATCA  Reverse: TCCAGATGAGCTCTCCAACCA  Probe: CAAAAACGCAAAGAAT | 77 | EF485029.1 | This publication |
| **GAPDH** | Forward: AAGTGGATATTGTCGCCATCAAT  Reverse: AACTTGCCATGGGTGGAATC  Probe: ACTACATGGTCTACATGTTTCAGTA (#) | 88 | AF097178 | adapted from [2] |
| **IFN-γ** | Forward: GGAGGACCTGTTCGTTAAGTTCTTT (#)  Reverse: TTTGCGCTGGACCTTCAGA  Probe: ATTCAGATTCCGGTAAATGA | 103 | NM_001081949 | adapted from [3] |
| **IL-1β** | Forward: CCGACACCAGTGACATGATGA  Reverse: TCCTCCTCAAAGAACAGGTCATTC  Probe: CTTACTGCAGCGGCAAT (#) | 64 | NM_001082526 | adapted from [4] |
| **IL-2** | Forward: CAAGAATCCCAAACTCTCCAAGAT  Reverse: TCGAGAGAAAGTTTTTTAGCATTTCC  Probe: CCCAAGAAGGCCACAGA (#) | 134 | NM_001085433 | adapted from [2] |
| **IL-4** | Forward: TCGTGCATGGAGCTGACTGTA  Reverse: CAGCCCTGCAGATTTCCTTTC (#)  Probe: GCCCGAAGAACACAGA (#) | 76 | NM_001082519 | adapted from [5] |
| **IL-5** | Forward: TGCTTCTGCATTTGAGTGTGCTA  Reverse: TGTTCATGGGACTTTCTACAGCAA  Probe: CTCTTGGAGCTGCCTAC (#) | 75 | NM_001082499 | adapted from [6] |
| **IL-6** | Forward: GATGCTTCCAATCTGGGTTCA (#)  Reverse: GCATAGTCTTGATGTTTTCCTTTTC (#)  Probe: ATCAGGCAGGTCTCCTG | 132 | NM_001082496 | adapted from [4] |
| **IL-6R** | Forward: GAAGGCGGCTGGTTCTGA  Reverse: CCTGGTAGCACGAATAGTTTCCA  Probe: TTCCGTGCAGCTCAG (#) | 63 | XM_005610076 | This publication |
| **IL-8** | Forward: GGGCCACACTGCGAAAACT (#)  Reverse: GCACAATAATCTGCACCCACTTT  Probe: GAAATCATTGTAAAGCTCG (#) | 97 | NM_001083951 | adapted from [7] |
| **IL-10** | Forward: GACATCAAGGAGCACGTGAACTC  Reverse: TGCTCCACTGCCTTGCTCTT  Probe: GAAATCATTGTAAAGCTCG (#) | 113 | NM_001082490 | adapted from [2] |

**Additional file 8: Nucleotide sequences of equine-specific primers used in real-time PCR assays** *(continued)*

| **Target** | **Primers and Probe (5'-3')** | **Product**  **size (bp)** | **GenBank**  **Accession** | **References** |
| --- | --- | --- | --- | --- |
| **IL-12** | Forward: ACCAGGCCCAGGAATGTTC  Reverse: TGACGGTCCTCAGCAGGTTT  Probe: TGCCTCAACCACTCC | 59 | NM_001082511 | This publication |
| **IL-13** | Forward: CCCTGGAGTCCCTGAGCAA  Reverse: CATCTTCCGCGTGTTTTGG  Probe: CCACCTGCAGTGCCAT (#) | 59 | NM_001143791 | This publication |
| **IL-17** | Forward: ATCGTGAAGGCGGGAATAGTAA  Reverse: TCGTTTTCCGGTTAAGGACG  Probe: ACAAGAACTTCCCTCAGAAT (#) | 112 | AY014959 | adapted from [8] |
| **IL-18** | Forward: GAAATCAACCTGTGTTTGAGGATATG  Reverse: TCACAGAGATGGTTACCGCTAGAC  Probe: ATTGTACAGACAACGCACCC | 126 | NM_001082512 | This publication |
| **IL-23** | Forward: CCCATATCCAGTGCGAGGAT  Reverse: CTTTGCAAGCAGGGCTGACT  Probe: TGTGATCCTGAAGGACT | 70 | NM_001082522 | This publication |
| **TGF-β** | Forward: TCCTGGCGCTACCTCAGTAAC  Reverse: TGACATCAAAGGACAGCCATTC  Probe: CTGCTGACCCCCAGCGACTCG | 70 | NM_001081849 | [9] |
| **TNF-α** | Forward: CTTCTGCCTGCTGCACTTTG (#)  Reverse: GGGCTACAGGCTTGTCACTTG (#)  Probe: CCAGACACTCAGATCAT | 134 | NM_001081819 | adapted from [3] |

*(#) represents modifications from previously published literature; ACTB, Actin beta; GUS, glucoronidase; GAPDH, Glyceraldehyde 3-phosphate dehydrogenase; IFN, Interferon; IL, interleukin; TGF, Transforming Growth Factor; TNF, tumor necrosis factor*

1. Coombs DK, Patton T, Kohler AK, Soboll G, Breathnach C, Townsend HGG, et al. Cytokine responses to EHV-1 infection in immune and non-immune ponies. Vet Immunol Immunopathol. 2006;111:109–16.

2. Colahan PT, Kollias-Baker C, Leutenegger CM, Jones JH. Does training affect mRNA transciption for cytokine production in circulating leucocytes? Equine Vet J. 2002;34:154–158.

3. Horohov DW, Breathnach CC, Sturgill TL, Rashid C, Stiltner JL, Strong D, et al. In vitro and in vivo modulation of the equine immune response by parapoxvirus ovis. Equine Vet J. 2008;40:468–72.

4. Saulez MN, Godfroid J, Bosman A, Stiltner JL, Breathnach CC, Horohov DW. Cytokine mRNA expressions after racing at a high altitude and at sea level in horses with exercise-induced pulmonary hemorrhage. Am J Vet Res. 2010;71:447–453.

5. Ainsworth DM, Appleton JA, Eicker SW, Luce R, Flaminio MJ, Antczak DF. The effect of strenuous exercise on mRNA concentrations of interleukin-12, interferon-gamma and interleukin-4 in equine pulmonary and peripheral blood mononuclear cells. Vet Immunol Immunopathol. 2003;91:61–71.

6. Davis MS, Malayer JR, Vandeventer L, Royer CM, McKenzie EC, Williamson KK. Cold weather exercise and airway cytokine expression. J Appl Physiol. 2005;98:2132–6.

7. Allen CA, Payne SL, Harville M, Cohen N, Russell KE. Validation of quantitative polymerase chain reaction assays for measuring cytokine expression in equine macrophages. J Immunol Methods. 2007;328:59–69.

8. Ainsworth DM, Wagner B, Erb HN, Young JC, Retallick DE. Effects of in vitro exposure to hay dust on expression of interleukin-17, -23, -8, and -1beta and chemokine (C-X-C motif) ligand 2 by pulmonary mononuclear cells isolated from horses chronically affected with recurrent airway disease. Am J Vet Res. 2007;68:1361–9.

9. Dahlgren LA, Mohammed HO, Nixon AJ. Temporal expression of growth factors and matrix molecules in healing tendon lesions. J Orthop Res. 2005;23:84–92.
